# Absence of Bcl-2 and Fas/CD95/APO-1 predicts the response to immunotherapy in metastatic renal cell carcinoma

**DOI:** 10.1038/sj.bjc.6603359

**Published:** 2006-10-10

**Authors:** R Maruyama, K Yamana, T Itoi, N Hara, V Bilim, T Nishiyama, K Takahashi, Y Tomita

**Affiliations:** 1 Division of Molecular Oncology, Department of Signal Transduction Research, Graduate School of Medical and Dental Sciences, Niigata University, Asahimachi 1-757, Niigata 951-8510, Japan; 2 Department of Urology, Yamagata University Faculty of Medicine, Iida-nishi 2-2-2, Yamagata 990-9585, Japan

**Keywords:** Bcl-2, Fas, renal cell carcinoma, immunotherapy

## Abstract

Immunotherapy is the only available treatment for metastatic renal cell cancer (RCC), but the response rate is only about 20% and the treatment is occasionally associated with severe adverse effects. Thus, the selection of patients with a high susceptibility to immunotherapy is needed; however, there is no promising molecular marker that can predict the response to immunotherapy for RCC. This study was carried out to elucidate the potential role of apoptosis-related molecules Bcl-2 and Fas, as well as apoptotic and proliferating indexes (AI, PI) as predictors of the susceptibility of metastatic RCC to immunotherapy. Immunohistochemical examination of tumour tissues from 40 patients with metastatic RCC undergoing postoperative immunotherapy after radical nephrectomy was performed. Patients with progressive disease (PD) after immunotherapy presented with decreased survival (*P*=0.006). Progressive disease correlated with higher PI in the primary lesion (*P*=0.0087). All primary tumours of CR or PR patients were negative for Bcl-2, whereas among NC+PD patients, 40.6% were positive for Bcl-2 (*P*=0.0373). Patients in whom the primary tumours were both Bcl-2- and Fas-negative showed significantly better responses to immunotherapy in comparison with the remaining group (*P*=0.0022). The Bcl-2 and Fas status of the primary lesion may become useful criteria for the selection of patients with metastatic RCC for immunotherapy.

Metastatic renal cell cancer (RCC) generally has a fatal outcome, despite conventional chemotherapy, radiotherapy and hormonal therapy. However, immunotherapy, such as the administration of interferons (IFNs) or interleukin-2 (IL-2) has shown promise, although the efficacy varies among cases and the total response rate is approximately 20%. Immunotherapy should be performed only in selected responders, because the treatment is sometimes accompanied by severe adverse effects. Patients with a good performance status (Atkins *et al*, 1993; Fyfe *et al*, 1995), prior nephrectomy (Atkins *et al*, 1993; Fyfe *et al*, 1995; Figlin *et al*, 1997; Rosenberg *et al*, 1998), metastases predominantly located in the lung (Atkins *et al*, 1993; Fyfe *et al*, 1995; Rosenberg *et al*, 1998) and only one site of metastatic disease (Negrier *et al*, 1998), with a clear cell histological variant (Wu *et al*, 1998) are more likely to exhibit a favourable response to immunotherapy. To date, however, no reliable molecular markers have been identified that can predict susceptibility to immunotherapy.

However, the mechanisms of RCC resistance to immunotherapy are not well defined. Some *in vitro* studies have shown that Fas (CD95/Apo1) promotes apoptosis in renal carcinoma cells by immune cells and Bcl-2 prevents it. Previously, we demonstrated the sensitisation of RCC cells to Fas-mediated apoptosis by IFN-*γ* (Tomita *et al*, 1996a) *in vitro*. This effect was attributed to the regulation of downstream caspases (Tomita *et al*, 2003). Cytokines, including IFNs, upregulate Fas in RCC cells (Nonomura *et al*, 1996), and thus increase the susceptibility to Fas-mediated apoptosis. In RCC cell lines, Bcl-2 reduction has been shown to be associated with increased sensitivity to anti-Fas (Hara *et al*, 2001). We found that Fas remarkably induced apoptosis in cells with low Bcl-2 levels compared with high Bcl-2 expressors (Tomita *et al*, 1996a). Furthermore, it has been recently shown that the downregulation of Bcl-2 sensitises IFN-resistant renal cancer cells to Fas (Kelly *et al*, 2004). Based on these studies, we considered that Bcl-2 may be associated with the resistance of RCC to immunotherapy *in vivo*.

Since 1987, we have accumulated specimens from about 500 RCC patients and followed their clinical course. We previously investigated several apoptotic factors, together with their signalling mechanism and reported the prognostic value of serum soluble Fas in RCC patients (Kimura *et al*, 1999). Furthermore, the frequent expression of Bcl-2 and the absence of p53 gene alterations were found in these RCC specimens (Tomita *et al*, 1996b). Most recently, we reported the relationship between Bcl-2 expression and good prognosis in RCC patients and demonstrated a significant difference in survival between Bcl-2-positive and -negative patients without metastasis (Itoi *et al*, 2004). Nevertheless, the role of Bcl-2 appeared to be equivocal in patients with metastatic disease. Thus, our interest focused on the detailed clinical course, including response to immunotherapy in addition to the expression of apoptosis-associated molecules Bcl-2 and Fas, Ki-67 cell proliferation activity, TUNEL apoptotic index in metastatic RCC, which remains not a rare clinical problem despite recent advances in diagnostic modalities and techniques.

We found that patients with metastatic RCC whose primary tumours were both Bcl-2- and Fas-negative demonstrated a better response to immunotherapy in clinical settings.

## MATERIALS AND METHODS

### Patients

Forty patients (35 men and five women) who underwent radical nephrectomy for RCC between 1991 and 2001 at our institution and presented with metastasis at the time of operation were recruited in this study (Table 1). Experiments were carried out with approval from the University's Ethical Committee. The histological diagnosis, grading and staging of the tumours were determined according to UICC TNM classification. Following nephrectomy, all patients were subjected to immunotherapy, including treatment with IFN-*α*, IFN-*γ* and/or IL-2, and some patients received combined treatment with low-dose anticancer drugs, 5-fluouracil or tegafur uracil, as presented in Table 1. The mean follow-up period was 39 months (range 2–150). We evaluated the response to immunotherapy according to the General Rule for Clinical And Pathological Studies on Renal Cell Carcinoma (April 1999, The 3rd Edition) published by the Japanese Urological Association, Japanese Society of Pathology and Japanese Radiological Society.

### Immunohistochemistry

Avidin–biotin immunoperoxidase staining of fresh-frozen tissue sections (5 *μ*m) of primary tumours in 28 cases and both primary and metastatic sites in 12 cases was performed as described previously (Tomita *et al*, 1993). The following primary monoclonal antibodies were used – anti-Bcl-2 clone 124 (1/100 dilution, Dako, Glostrup, Denmark), anti-Fas clone UB-2 (1/100 dilution, Medical Bioscience Laboratory, Nagoya, Japan) and anti-human Ki-67 antigen clone MIB-1 (1/100 dilution, Dako). The specificity of each antibody to Bcl-2 and Fas had been confirmed previously (Itoi *et al*, 2004; Yamana *et al*, 2005). Negative controls were prepared by omission of the primary monoclonal antibodies.

### Evaluation of immunostaining

The staining results were evaluated independently by two observers, neither of whom had prior knowledge of the clinical or pathological data. There were no inter- and intra-sample fluctuations in terms of staining intensity. Immunoreactivity for Bcl-2 and Fas was graded according to the criteria as follows: strong, more than 50% positively stained tumour cells; moderate, 10–50%; weak, less than 10%; negative, no positive tumour cells in all fields. Tumour specimens showing weak staining were considered negative. Those with moderate and strong staining were considered positive. The proliferation index (PI) was expressed as the percentage of Ki-67-positive cells among the tumour cells, with at least 1000 cells counted in several fields for each section, as reported previously (Itoi *et al*, 2004).

### Detection of apoptosis

For TUNEL staining, an *In Situ* Cell Death Detection Kit (Takara, Japan) was used according to the manufacturer's instructions. Cells were considered TUNEL-positive when brown reactivity was detected in the nuclei. The apoptosis index (AI) was expressed as the percentage of TUNEL-positive cells among the tumour cells, with at least 1000 cells counted in several fields for each section, as reported previously (Itoi *et al*, 2004).

### Statistical analysis

Statistical analysis was performed using StatView 5.0 for Macintosh (Abacus Concepts, Berkeley, CA, USA). The *χ*^2^ and Fisher's exact test were used to assess the correlation between the expression and clinicopathological parameters. The Kaplan–Meier method was used to determine survival, and the log-rank test was used to compare curves. *P*-values less than 0.05 were considered statistically significant.

## RESULTS

### Clinical course of the patients

**Figure 1:**
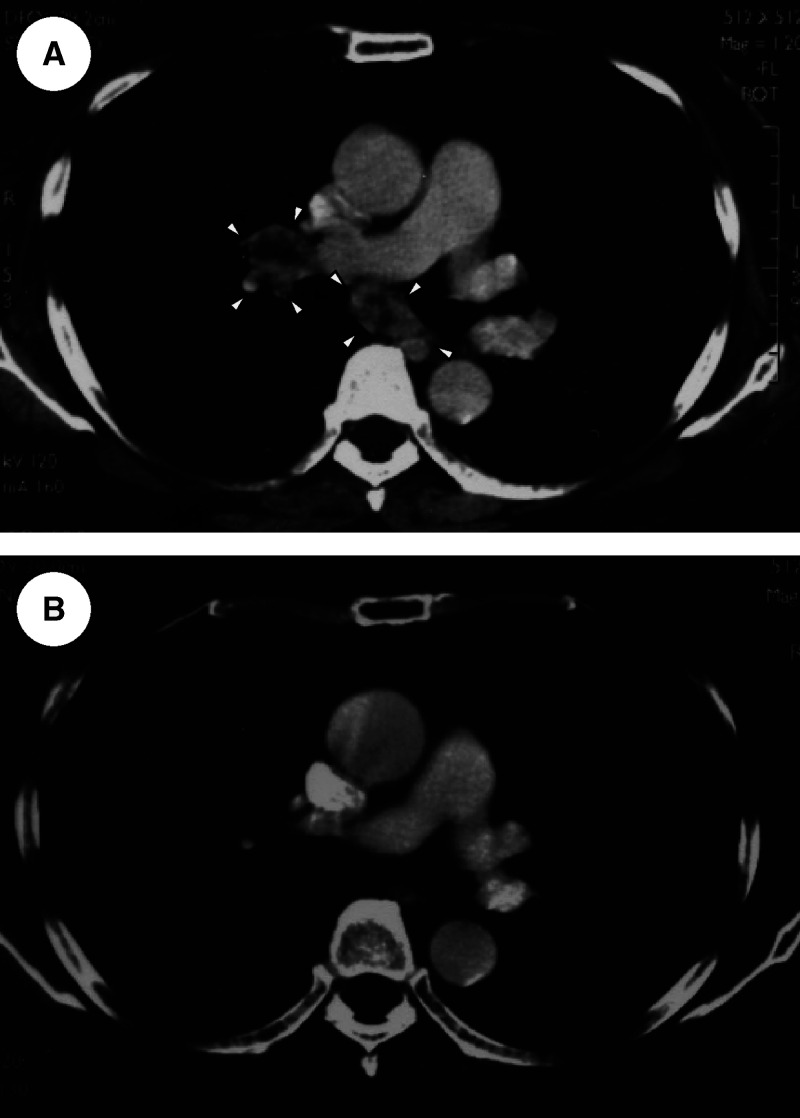
(**A**) Patient No. 8 chest CT shows mediastinal lymph nodes at diagnosis (arrrowheads). (**B**) Mediastinal lymph node swelling disappeared after immunotherapy (CR).

Among 40 patients, a clinical response was observed in eight patients (20.0%) (Figure 1 and Table 2) among which three showed a complete response (CR) and five showed a partial response (PR). The metastasis sites in responders were as follows: lung in four, and one each in the following sites: brain and lung (which was removed surgically before the start of immunotherapy and the patient subsequently achieved CR), bone and lung (as above), liver only, and lung and mediastinal lymph nodes (Figure 1). Complete responses and/or PRs continued from 2 to 49 months. The presence of metastases in just the lung *vs* other locations correlated with longer survival (*P*=0.0333).

**Figure 2:**
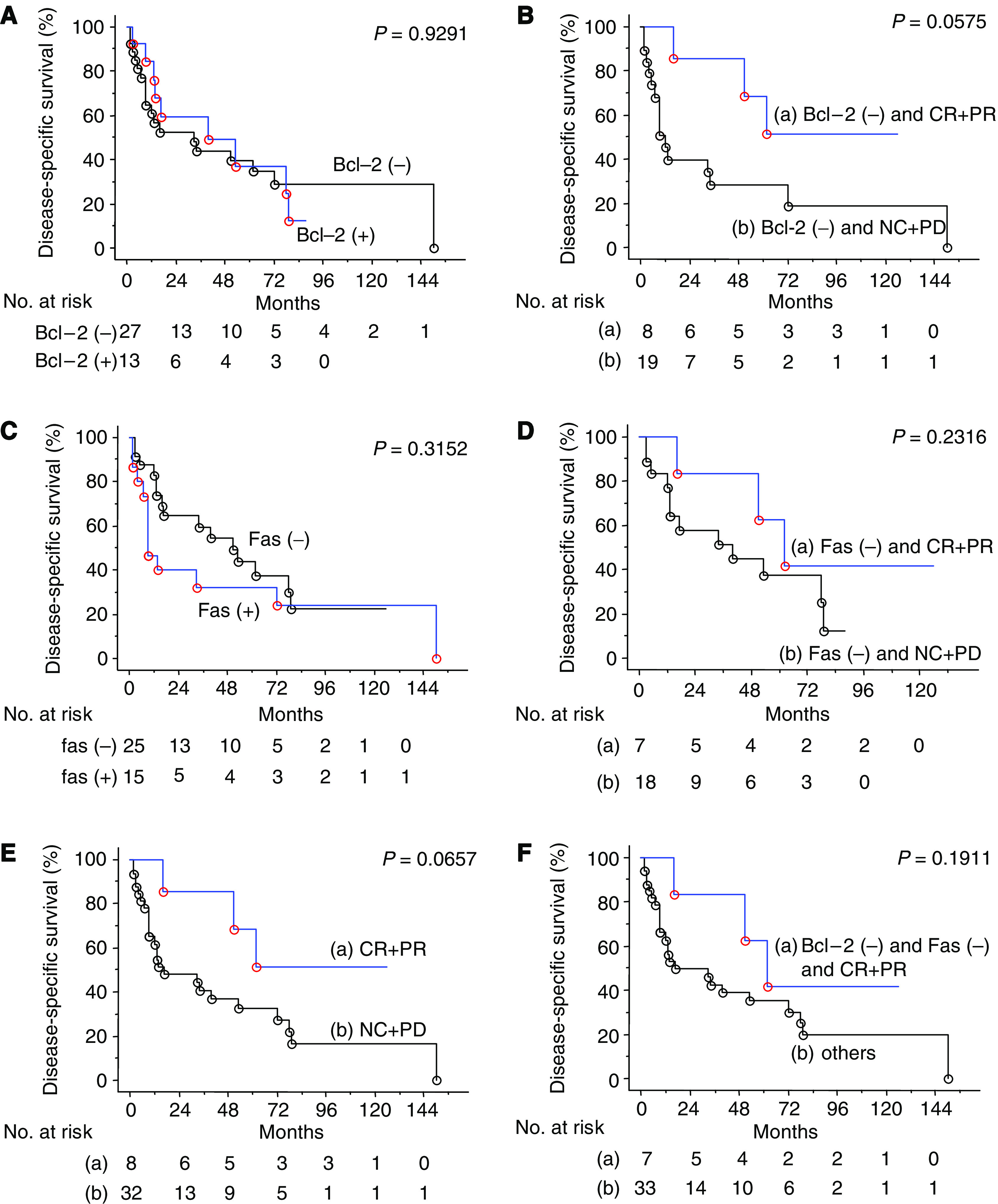
(**A**) Disease-specific survival of all cases according to Bcl-2 expression. (**B**) Disease-specific survival in Bcl-2-negative cases only according to the response to immunotherapy. (**C**) Disease-specific survival of all cases according to Fas expression. (**D**) Disease-specific survival in Fas-negative cases only according to the response to immunotherapy. (**E**) Comparison of disease-specific survival between CR+PR *vs* NC+PD. (**F**) Comparison of disease-specific survival between Bcl-2 and Fas-negative cases in CR+PR *vs* others.

Patients with progressive disease (PD) presented with decreased disease-specific survival (*P*=0.006) (Figure 2E); however, no difference in survival was detected between the responders (CR+PR) and the non-responders (NC+PD) (*P*=0.0657) (Figure 2E).

### Expression of Bcl-2

**Figure 3:**
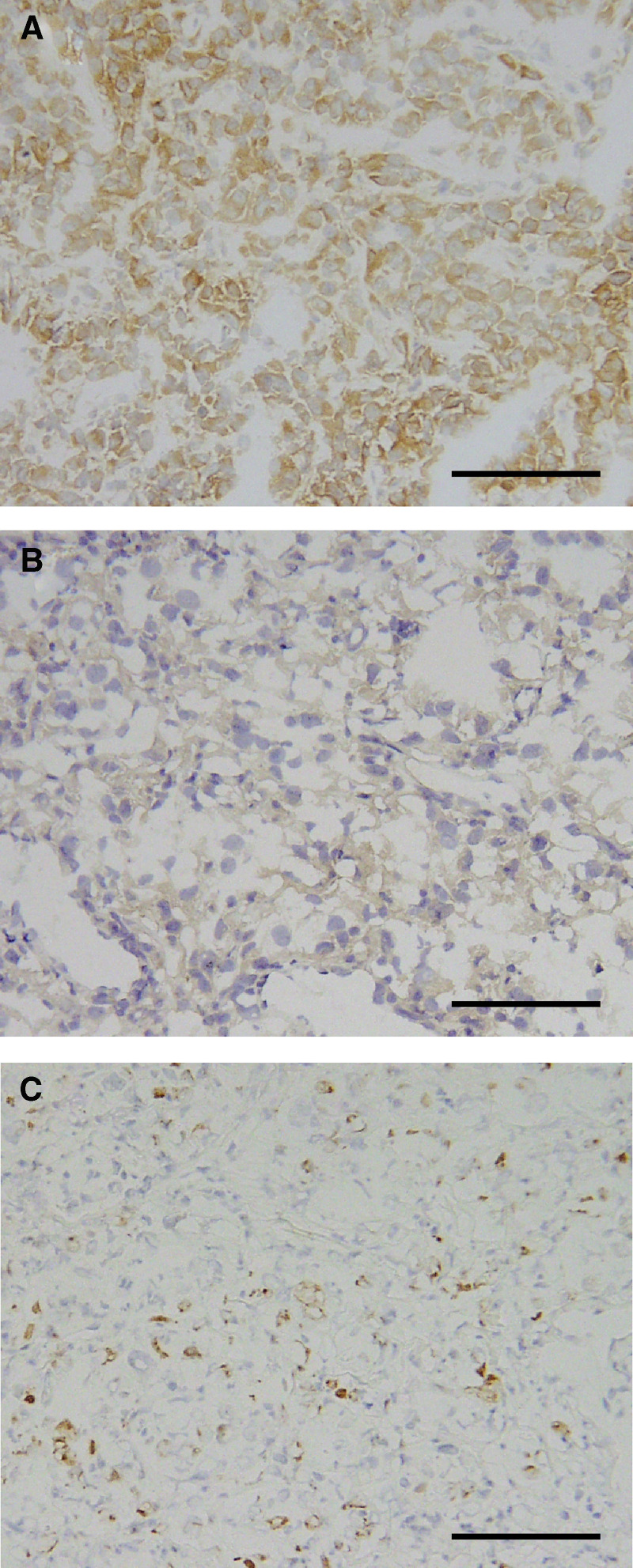
Immunohistochemical staining of (**A**) Bcl-2 (No. 300), (**B**) Fas (No.84), (**C**) Ki-67 (No. 242). Scale bars represent 100 *μ*m.

Bcl-2 was detected in the cytoplasm of cancer cells (Figure 3A). Bcl-2 was expressed in 13 of 40 (32.5%) primary specimens and two of 12 (16.7%) metastatic lesions. Bcl-2 expression was not related to the T stage or the tumour grade (Table 3). Bcl-2 staining was positive in 13 of 32 (40.6%) primary tumours from patients with no response (NC+PD) to immunotherapy and in 0 (0%) of eight responders (CR+PR) (*P*=0.0373) (Tables 2, 3). In addition, four of the five responders examined were negative for Bcl-2 at metastatic sites (Table 2). In this series of patients, there was no correlation between Bcl-2 expression and disease-specific survival (Figure 2A). In Bcl-2-negative cases, responders to immunotherapy showed a better prognosis than non-responders (*P*=0.0575) (Figure 2B). Nevertheless, there was no difference in the disease-specific survival between responders and non-responders. The expression of Bcl-2 was not correlated to Fas; however, Fas expression was significantly higher in the selected group of Bcl-2(−) non-responders (91.7%) than responders (8.3%) (*P*=0.0433).

### Expression of Fas

Fas was detectable on the cell membranes and within the cytoplasm of cancer cells (Figure 3B). It was expressed in 15 of 40 (37.5%) primary specimens and three of 12 (25%) metastatic lesions. Fas expression was not related to the T stage or the tumour grade (Table 3). Although there was no correlation between responders to immunotherapy (CR+PR) and Fas expression, patients with PD after immunotherapy expressed Fas more frequently in primary tumours than other patients (CR+PR+NC) (*P*=0.0484) (Table 3). Among the eight responders, only one primary tumour expressed Fas, and all five metastatic tumours available from responders were negative for Fas, including a metastatic tumour from a patient with positive Fas on the primary RCC (Table 2). The disease-specific survival of patients characterised according to Fas staining was not different (Figure 2C); responders in the Fas(−) group tended to have longer survival, although it was not statistically significant (Figure 2D). Both the Bcl-2- and Fas-negative status correlated with the response (CR+PR) to immunotherapy (*P*=0.0022) (Table 4), although this had no effect on survival (Figure 2F).

### Detection of cell proliferation and apoptosis

Ki-67 was expressed in the nuclei of cancer cells (Figure 3C). Proliferation index ranged from 0.66 to 20.52% (mean 5.28%). Proliferation index was significantly higher in G3 than G1-2 (*P*=0.0287) and Bcl-2-negative *vs* -positive cases (*P*=0.0390) (Table 5). Although there was no correlation between PI and response to immunotherapy (CR+PR), higher PI correlated with decreased survival (*P*=0.0189) and patients with PD presented with significantly higher PI in the primary tumour (*P*=0.0087). Apoptosis index ranged from 0 to 2.74% (mean 0.56%). No clinicopathological parameter correlated with AI.

## DISCUSSION

It is feasible that Bcl-2 represses immunotherapy-induced apoptosis in RCC cells, as all primary tumours from responders to immunotherapy were negative for Bcl-2 in this study; however, a clinical response was observed in only eight of 27 (29.6%) Bcl-2-negative cases. Therefore, we subclassified RCC patients into three groups according to the Bcl-2 status and the response to immunotherapy: negative Bcl-2 expression and good response to immunotherapy, negative Bcl-2 expression and no response to immunotherapy, and positive Bcl-2 expression (all non-responders). Bcl-2-positive cancers presented with lower PI determined with Ki-67 (*P*=0.039) (Table 5), which can explain the better prognosis in non-metastatic cases reported previously (Itoi *et al*, 2004). Bcl-2 has been found to delay entry into the cell cycle and repress cell proliferation in addition to its antiapoptotic action (Linette *et al*, 1996; Mazel *et al*, 1996; O'Reilly *et al*, 1996). Furthermore, the antiproliferative effect of Bcl-2 is separate from the antiapoptotic effect (Uhlmann *et al*, 1996; Huang *et al*, 1997), and became undetectable, whereas antiapoptotic activity persists as cancer progresses (Furth *et al*, 1999); however, there was no correlation between the Bcl-2 expression and prognosis in this study (Figure 2A). This can be explained by the fact that all patients in this group had metastasis at the time of surgery. Moreover, the cancer cells in this study were challenged with exposure to immune attack, which should presumably induce apoptosis. In this setting, the presence of Bcl-2 prevented apoptosis and ultimately shortened patient survival. So far, Bcl-2(−) responders have showed better survival (Figure 2B), confirming this hypothesis.

We propose the following model based on these findings. During the initial proliferative process, Bcl-2-positive tumour cells have both antiapoptotic and antiproliferative activity. As the disease progresses, antiproliferative activity is overcome by the activation of multiple tumour-specific intracellular pathways, whereas the antiapoptotic effect persists and renders tumour cells resistant to apoptosis by immune cell attack. In contrast, Bcl-2-negative tumours are sensitive to immunotherapy-triggered apoptosis. We hypothesised that Bcl-2-negative tumour cells with higher proliferative activity, as demonstrated by Ki-67, may overcome the apoptotic effect of immune cells in this case. Although PI was not significantly higher in Bcl-2(−) responders *vs* non-responders, PI was significantly higher in PD patients after immunotherapy than others (*P*=0.0087). Thus, patients with high PIs are considered inappropriate candidates for immunotherapy, although a low PI does not necessarily guarantee a clinical response.

In the Bcl-2(−) non-responders subgroup, other factors can oppose apoptosis. Fas, the main target of cytotoxic immune cells, and the Fas pathway status may be responsible for such distinction. Although the expression of Bcl-2 was not correlated to Fas, Fas expression was significantly higher in the non-responders group Bcl-2(−) than responders (*P*=0.0433). Furthermore, PD patients with immunotherapy expressed Fas more frequently than others (CR+PR+NC) (*P*=0.0484) (Table 3). These findings conflict with the generally accepted view that Fas mediates immune cell-triggered apoptosis; however, Fas stimulation can be antagonised by the expression of non-functional Fas splicing variants, Decoy receptor 3, the presence of soluble Fas, by Fas gene mutations, or by the expression of downstream inhibitors, such as XIAP or Bcl-2. Renal cell cancer has been reported to harbour mutations in exons 7 and 9 of the Fas gene (Takayama *et al*, 2002). Indeed, we have previously demonstrated that RCC patients have elevated levels of soluble Fas, which was an independent negative prognostic factor (Kimura *et al*, 1999). In addition to inducing apoptosis, Fas can also promote cell growth or differentiation (Mapara *et al*, 1993; Owen-Schaub *et al*, 1994; Budd, 2002). From this point of view, it can be explained why Bcl-2-negative and Fas-negative patients had a better response to immunotherapy in metastatic RCC.

Bcl-2 and Fas can become useful predictive markers for response to immunotherapy in metastatic RCC patients in clinical settings, and PI can be an additional factor to predict the response to immunotherapy. Although larger trials are necessary, the results of this pilot study suggest that patients with Bcl-2-negative and Fas-negative RCC could be appropriate candidates for immunotherapy.

## Figures and Tables

**Table 1:** Patient characteristics

		**(%)**
Mean age (range) years	59.4 (37–83)	
Male/female	35/5	
		
*T stage*		
T1	7	17.5
T2	5	12.5
T3	23	57.5
T4	5	12.5
		
*Grade*		
1	9	22.5
2	26	65.0
3	5	12.5
		
*Histological type*		
Clear cell	38	95.0
Papillary	2	5.0
		
*Metastatic site*		
Lung (lung mets only)	30 (18)	
Other site	22	
		
*Treatment*		
IFN-*α*	15	37.5
IFN-*α*+5FU	5	12.5
IFN-*γ*	8	20.0
IL-2	4	10.0
IL-2+IFN-*α*+TU	8	20.0

5FU=5-fluorouracil; IFN=interferon; IL=interleukin; TU=tegafur uracil.

**Table 2:** Characteristics of responders to immunotherapy

								**Expression^a^**					
**No.**	**Age**	**Sex**	**T**	**N**	**G**	**Histological type**	**Metastatic site**	**Bcl-2**	**Fas**	**Treatment**	**Best response (duration, mos)**	**Follow up (mos)**	**Status at last follow-up**
1	56	M	3b	0	1	Clear cell	Lung	−/−	−/−	IL-2	PR (3)	14	DOD
2	37	M	3a	1	2	Clear cell	Lung	−/+	−/−	IL-2	PR (9)	50	DOD
3	61	M	1a	0	1	Clear cell	Lung	−/−	−/−	IFN-*α* +5FU	PR (7)	106	AWD
4	68	M	3a	0	2	Clear cell	Lung	−/−	+/−	IL-2	PR (10)	125	NED^b^
5	66	F	1b	0	2	Clear cell	Lung	−/−	−/−	IFN-*α* + (5FU → TU)	PR (49)	100	AWD
6	54	M	3a	0	3	Papillary	Liver	−/NA	−/NA	IFN-*α*	PR (2)	2	DOC
7	53	M	3a	0	2	Clear cell	Lung	−/NA	−/NA	IFN-*α*	CR (33)	62	DOD
8	62	F	3b	2	3	Clear cell	Lung, LN	−/NA	−/NA	IL-2+IFN-*α*+TU	CR (28)	30	NED

AWD=alive with disease; CR=complete response; DOC=died of other causes; DOD=died of disease; 5FU=5-fluorouracil; IFN=interferon; IL=interleukin; LN=mediastinal lymph node; mos=months; NA=not available; NED=no evidence of disease; PR=partial response; TU=tegafur uracil.

a Expression of primary site/metastatic site.

b No evidence of disease after resection of lung metastasis.

**Table 3:**
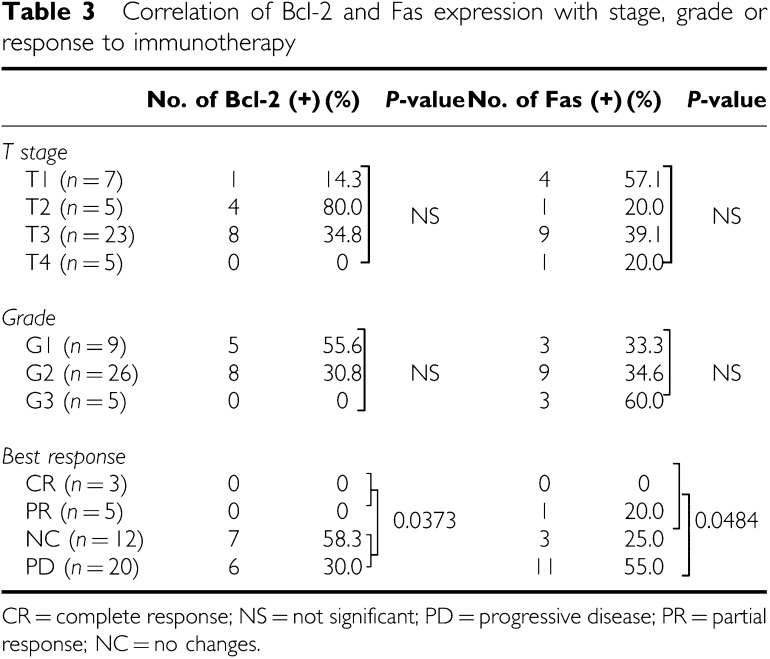
Correlation of Bcl-2 and Fas expression with stage, grade or response to immunotherapy

**Table 4:** Correlation of Bcl-2 and Fas expression with response to immunotherapy

	**Response (%)**		
	**CR or PR**	**NC or PD**	***P*-value**
Bcl-2(−) and Fas(−) (*n*=15)	7 (46.7)	8 (53.3)	0.0022
Bcl-2(+) and/or Fas(+) (*n*=25)	1 (4.0)	24 (96.0)	

CR=complete response; PD=progressive disease; PR=partial response; NC=no changes.

**Table 5:** Correlation of AI and PI with stage, grade, response to immunotherapy and expression of Bcl-2 and Fas

	**AI (%, mean±s.d.)**	***P*-value**	**PI (%, mean±s.d.)**	***P*-value**
*T stage*				
T1,2	0.675 (±0.693)	NS	5.002 (±3.189)	NS
T3,4	0.511 (±0.558)		5.403 (±4.316)	
				
*Grade*				
G1,2	0.603 (±0.625)	NS	4.629 (±3.179)	0.0287
G3	0.260 (±0.174)		9.858 (±6.184)	
				
*Response*				
CR+PR	0.380 (±0.281)	NS	6.635 (±6.127)	NS
NC+PD	0.605 (±0.648)		4.945 (±3.288)	
				
Bcl-2(+)	0.774 (±0.942)	NS	3.978 (±3.865)	0.0390
Bcl-2(−)	0.457 (±0.303)		5.911 (±3.943)	
				
Fas(+)	0.626 (±0.667)	NS	5.442 (±2.796)	NS
Fas(−)	0.524 (±0.562)		5.187 (±4.593)	

AI=apoptosis index; CR=complete response; NS=not significant; PD=progressive disease; PI=proliferation index; PR=partial response; s.d.=standard deviation; NC=no changes.
